# A Parameter Reduction-Based Decision-Making Method with Interval-Valued Neutrosophic Soft Sets for the Selection of Bionic Thin-Wall Structures

**DOI:** 10.3390/biomimetics9040208

**Published:** 2024-03-29

**Authors:** Honghao Zhang, Lingyu Wang, Danqi Wang, Zhongwei Huang, Dongtao Yu, Yong Peng

**Affiliations:** 1 Key Laboratory of High Efficiency and Clean Mechanical Manufacture (Ministry of Education), School of Mechanical Engineering, Shandong University, Jinan 250061, China; honghao_zhang@sdu.edu.cn (H.Z.); 202214329@mail.sdu.edu.cn (L.W.); dongtaoyu2022@mail.sdu.edu.cn (D.Y.); 2 Key Laboratory of Transportation Industry for Transport Vehicle Detection, Diagnosis and Maintenance Technology, Jinan 250061, China; 3 College of Automotive and Mechanical Engineering, Changsha University of Science and Technology, Changsha 410114, China; 4 Key Laboratory of Traffic Safety on Track of Ministry of Education, School of Traffic and Transportation Engineering, Central South University, Changsha 410083, China; yong_peng@csu.edu.cn

**Keywords:** bio-inspired structures, interval-valued neutrosophic soft sets, parameter reduction, multi-criteria decision making, energy absorbing devices

## Abstract

Bio-inspired thin-wall structures with excellent mechanical properties, high-energy absorption capabilities, and a desirable lightweight level have been extensively applied to the passive safety protection of transportation and aerospace. Collaboration matching and the selection of optional structures with different bionic principles considering the multiple attribute evaluation index and engineering preference information have become an urgent problem. This paper proposes a parameter reduction-based indifference threshold-based attribute ratio analysis method under an interval-valued neutrosophic soft set (IVNS-SOFT) to obtain the weight vector of an evaluation indicator system for the selection of bionic thin-wall structures, which can avoid the problem of an inadequate subjective evaluation and reduce redundant parameters. An IVNS-SOFT-based multi-attributive border approximation area comparison (MABAC) method is proposed to obtain an optimal alternative, which can quantify uncertainty explicitly and handle the uncertain and inconsistent information prevalent in the expert system. Subsequently, an application of five bio-inspired thin-wall structures is applied to demonstrate that this proposed method is valid and practical. Comparative analysis, sensitivity analysis, and discussion are conducted in this research. The results show that this study provides an effective tool for the selection of bionic thin-wall structures.

## 1. Introduction

**Figure 1:**
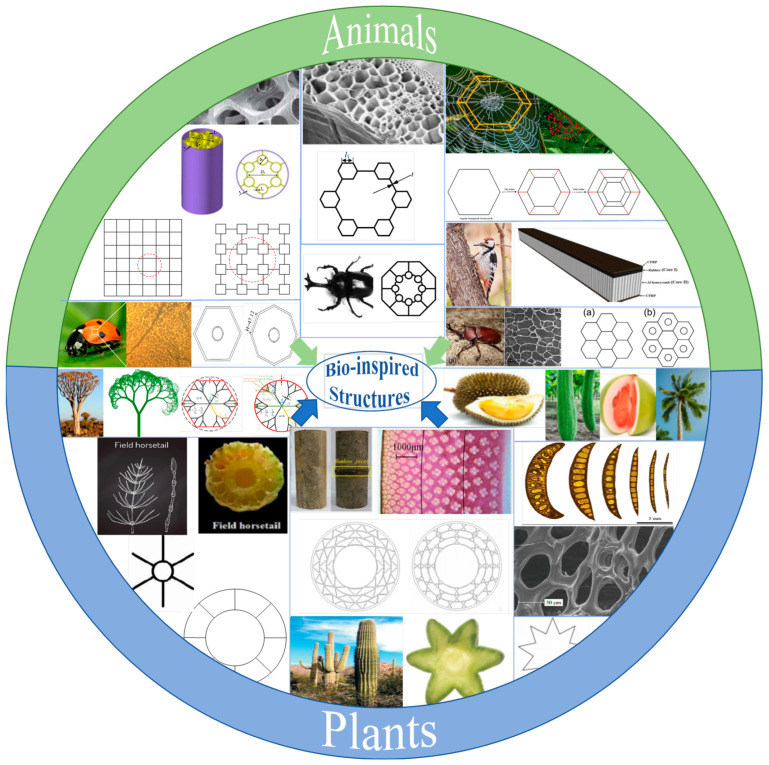
Bio-inspired thin-walled structures for energy absorption in the literature.

Inspired by biological structures in nature, with excellent characteristics of organisms in absorbing and dissipating energy, bio-inspired thin-walled structures have been proven to provide significant improvements in energy absorption capacity over conventional structures, e.g., pomelo fruits have a unique spongy mesocarp layer that can dissipate up to 80 J of energy from a free-fall test without causing significant external damage to the rind. Nuts also have excellent impact and puncture resistance, such as coconut shell and macadamia nut shell. Similarly, durian shells with special spines absorb impact energy well when the durian fruit falls and hits the ground. Lessons in high energy absorption can also be learned from animals. A beetle’s forewing can withstand a puncture force of up to 23 N, which is much higher than the beetle’s fighting strength, as shown in Figure 1. At present, a variety of structures have been widely considered and applied, including honeycomb structures, column structures, foam, porous materials, and hierarchical structures. Hierarchical structures have attracted extensive interest in engineering applications because of their excellent mechanical properties [1].

Organisms can form hierarchical structural materials with functional gradients, possessing load-bearing and supportive capabilities, providing design inspiration for bionic thin-walled structures that can realize unprecedented structural properties and functions [2]. Engineers have designed some lightweight bionic thin-walled structures inspired by nature [3,4,5], e.g., a Morpho wing-inspired thin-walled structure was proposed, which had greater specific energy absorption (SEA) and crush force efficiency (CFE) [6]. A new hexagonal honeycomb structure was inspired by the thickness gradient structure and the concept of hierarchy, which made the deformation mode more regular and stable [7]. A second hierarchical circular tube that possessed significant energy absorption properties by adding self-similar sub-circles at the junctions of the primary ribs was found [8]. A new hierarchical square thin-walled structure was constructed by replacing each vertex with a miniature cell to form a strict self-similarity [9]. Li et al. [10] replaced each node of the thin-walled hexagon with a small hexagon to obtain a fractal-like hexagonal tube with a self-similar cellular structure. Other kinds of self-similar structures have also been investigated, e.g., the improvement of the hexagonal honeycomb structure using a spider web topology, with the spider web hierarchy slowing down the onset of instability with high strength and toughness [11,12]. In summary, bionic structures are currently widely applied in automobiles, ships, airplanes, trains, and other industrial applications and, therefore, require different bionic design philosophies to meet specific application requirements.

However, different design philosophies mean that each bionic design functions differently [13]. The selection and determination of an optimal alternative usually need to consider multi-attributes, e.g., total energy absorption (EA), initial peak force (PCF), and the engineering requirements for specific application conditions, which can be regarded as a multi-criteria decision-making (MCDM) problem. 

At present, many MCDM methods have been designed and applied to solve the problem of the selection of thin-walled structures, e.g., the DEMATEL (Decision-Making Trial and Evaluation Laboratory)–TODIM(Tomada de Decisao Interativa Multicriterio) method [8], the GRA (Grey Relational Analysis) method [14], the COPRAS (Combination of Weighted Scores Method Based on Ratio Analysis) method [15,16], and the TOPSIS (Technique for Order Preference by Similarity to an Ideal Solution) method [17,18,19]. In addition, some effective MCDM methods are also used to solve similar selection problems, e.g., the indifference threshold-based attribute ratio analysis (ITARA) method [20,21], MABAC method [22] and the interval-value neutrosophic set (IVNS)–MABAC method [23,24]. Since the advent of the fuzzy set theory [25], researchers have started to use it to solve uncertainty problems, e.g., based on the fuzzy set theory, interval fuzzy sets [26], spherical-Z fuzzy sets [27], interval 2-tuple q-rung orthopair fuzzy sets [28] and soft sets [29] proposed. Nowadays, soft sets are increasingly studied and widely used in various fields, e.g., soft sets with neutrosophic sets [30,31], single-valued neutrosophic soft sets [32], and interval-valued neutrosophic soft sets [33]. 

As mentioned above, current research mainly focuses on the design and performance analysis of bionic thin-wall structures but rarely carries out structural selection research. Thus, this paper focuses on a parameter reduction-based decision-making method with interval-valued neutrosophic soft sets for the selection of bionic thin-wall structures. Compared with previous studies, the novelty and contributions of this study are summarized as follows: (1) a distance-based IVNS-SOFT parameter reduction method is proposed to reduce redundant parameters while retaining decision-making capability; (2) a hybrid MCDM method that combines IVNS-SOFT-based ITARA and MABAC is proposed, which takes into account the greater hesitancy of decision makers and the uncertainty of the decision environment, resulting in more accurate and effective decisions and results; and (3) empirical application and analysis, which are conducted to demonstrate that this proposed method is valid and practical.

The rest of this paper is organized as follows. Section 2 introduces the relevant concepts and definitions of IVNS-SOFT. Section 3 describes the interval-valued neutrosophic soft set-based MABAC decision-making method proposed in this paper and its main steps. Section 4 presents a case study of bio-inspired thin-walled tubes. Sensitivity analysis and comparative analysis of other methods are used to validate the scientific validity of the IVNS-SOFT-MABAC method. Section 5 summarizes the results and findings of the study and presents future perspectives.

## 2. Preliminary

In this section, the definitions and operations of IVNS-SOFT are shown, which combines IVNS and the soft set.

**Definition** **1** [34]**.** *Let U be an initial set, with a type of element in U denoted by x. An IVNS A in U is concluded by a true affiliation function T_A_(x), an uncertainty affiliation function I_A_(x), and a false affiliation function F_A_(x). Then, IVNS A can be expressed by Equation (S1)*.

**Definition** **2** [29]**.** *P(U) is the power set of U; E is a set of all parameters and* X⊆E*, as shown in Equation (S2). As a result, a soft set F_X_ over U is a set defined by a function representing a mapping, as shown in Equation (S3)*.

**Definition** **3** [34]**.** *IVNS(U) denotes the set of all IVNSs of U and E as a set of parameters that describe the elements of U and A⊆E. The pair (F, A) is called an IVNS-SOFT over U.*

(1) $${F(e)=\{(x,\;[T}_{F_{e}}^{L}{(x),\;T}_{F_{e}}^{U}{(x)],\;[I}_{F_{e}}^{L}{(x),\;I}_{F_{e}}^{U}{(x)],\;[F}_{F_{e}}^{L}{(x),F}_{F_{e}}^{U}(x)]):\;x\in U,\;e\;\in \;E\}$$

*where* $T_{Fe}^{L}{(x),\;T}_{Fe}^{U}\left(x\right)$ *are denoted as x with attribute e belonging to the lower and upper limit boundaries of the F(U) truth affiliation function, respectively.*$\;I_{Fe}^{L}{(x),\;I}_{Fe}^{U}\left(x\right)$ *and* $F_{Fe}^{L}{(x),\;F}_{Fe}^{U}\left(x\right)$ *are similar to the above*.

**Definition** **4** [33]**.** *The complement of (F, A), denoted by (F, A)^c^, is IVNS-SOFT over U and is defined as (F, A)^c^ = (F^c^, A), where F^c^: A → IVNS(U) is defined by Equation (S4).*

**Definition** **5** [33]**.** *Let (F, A), (G, B)* ∈ *IVNS-SOFT(U). If there are two IVNS-SOFTs, A and B*, *respectively, for all u* ∈ *U and A*$\;\tilde{\subseteq }\;$*B, then (F, A) is an IVNS-SOFT subset of (G, B), denoted by (F, A)* $\;\hat{\subseteq }$ *(G, B)*.

**Definition** **6** [35]**.** *Let (F, A) and (G, B) be two IVNS-SOFTs over a common universe set U. The union of (F, A) and (G, B) is denoted by* $\left(F,\;A\right)\tilde{\cup }\left(G,\;B\right)=(H,\;C)$*, where* C=A ∪ B *and the true affiliation function, uncertainty affiliation function and false affiliation function of (H, C) are shown in Equation (S5)*.

**Definition** **7** [35]**.** *Let*${\;x}_{11}{=([T}_{11}^{L}{,\;T}_{11}^{U}{],\;[I}_{11}^{L}{,\;I}_{11}^{U}{],\;[F}_{11}^{L}{,\;F}_{11}^{U}])\;$ *and* $x_{12}{=([T}_{12}^{L}{,\;T}_{12}^{U}{],\;[I}_{12}^{L}{,\;I}_{12}^{U}{],\;[F}_{12}^{L}{,\;F}_{12}^{U}])$ *be two interval-value neutrosophic soft numbers (IVNSNs) and ω > 0; then, the operations for the IVNSNs are defined as shown in Equations (S6)–(S9)*.

**Definition** **8** [36]**.** *A score function S of an IVNSN can be represented as follows in Equation (S10). The score function refers to a function that maps input values to a score and is used to combine the values of attributes into an overall score.*

**Definition** **9** [36]**.** *An accuracy function H(x_11_) is defined as shown in Equation (S11). The accuracy function is a function that accurately calculates or describes some mathematical object or relationship.*

**Definition** **10** [36]**.** *Let*${\;x}_{11}{=([T}_{11}^{L}{,\;T}_{11}^{U}{],\;[I}_{11}^{L}{,\;I}_{11}^{U}{],\;[F}_{11}^{L}{,\;F}_{11}^{U}])\;$ *and* $x_{12}{=([T}_{12}^{L}{,\;T}_{12}^{U}{],\;[I}_{12}^{L}{,\;I}_{12}^{U}{],\;[F}_{12}^{L}{,\;F}_{12}^{U}])$ *be two IVNSNs, let S(x_11_) and S(x_12_) be the scores, and* ${H(x}_{11})$ *and* ${H(x}_{12})$ *be the accuracy function*.

If${\;S(x}_{11}{)\;<\;S(x}_{12})$, then ${\;x}_{11}<x_{12}$.

If${\;S(x}_{11}{)=S(x}_{12})$, then, (1) if$\;{H(x}_{11}{)=H(x}_{12})$, then${\;x}_{11}=x_{12}$; (2) if${\;H(x}_{11}{)\;<\;H(x}_{12})$, then${\;x}_{11}<x_{12}$.

**Definition** **11** [35]**.** *Let* $x_{1j}\;\left(j=1,2,\cdots ,n\right)\;$ *be a series of the IVNSNs, and*$\;\omega ={\left(\omega_{1},\omega_{2},\cdots ,\omega_{n}\right)}^{T}$ *be the weight vector of* $x_{1j}=\left(j=1,2,\cdots ,n\right)$*, then an interval-value neutrosophic soft-weighted geometric (INSWG) operator can be seen as a mapping INSWG, as shown in Equation (S12)*.

**Definition** **12** [37]**.** *Let* $x_{11}{=([T}_{11}^{L}{,\;T}_{11}^{U}{],\;[I}_{11}^{L}{,\;I}_{11}^{U}{],\;[F}_{11}^{L}{,\;F}_{11}^{U}])\;$ *and* $x_{12}{=([T}_{12}^{L}{,\;T}_{12}^{U}{],\;[I}_{12}^{L}{,\;I}_{12}^{U}{],\;[F}_{12}^{L}{,\;F}_{12}^{U}])$ *be two IVNSNs, then the Euclidean distance between x_11_ and x_12_ can be defined, as shown in Equation (S13)*.

**Definition** **13** [37]**.** $P=\{\langle {[T}_{P}^{L}{(x}_{m}{),\;T}_{P}^{U}{(x}_{m}{)],\;[I}_{P}^{L}{(x}_{m}{),\;I}_{P}^{U}{(x}_{m}{)],\;[F}_{P}^{L}{(x}_{m}{),\;F}_{P}^{U}{(x}_{m})]\rangle \}$ *and* $Q=\{\langle {[T}_{Q}^{L}{(x}_{m}{),\;T}_{Q}^{U}{(x}_{m}{)],\;[I}_{Q}^{L}{(x}_{m}{),\;I}_{Q}^{U}{(x}_{m}{)],\;[F}_{Q}^{L}{(x}_{m}{),\;F}_{Q}^{U}{(x}_{m})]\rangle \}$ *are two IVNS-SOFTs. Then, the standard Euclidean distance between P and Q can be calculated as shown in Equation (S14)*.

## 3. Methods Section

In this section, IVNS-SOFT is invoked to describe the expert’s opinion, which can better represent the degree of uncertainty and ambiguity in the expert’s decision making. The parameter reduction algorithm, ITARA method, and MABAC method based on the IVNS-SOFT theory are proposed. Some definitions of IVNS-SOFT given in Section 2 are used in the computation of all the above methods.

### 3.1. The Framework of the Proposed Solution Methodology

**Figure 2:**
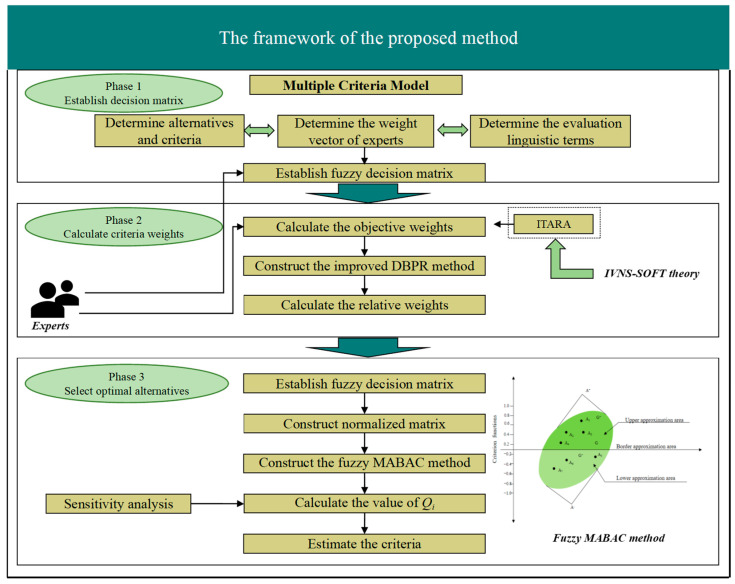
The framework of the proposed method.

The framework of the MCDM method proposed in this paper for the optimal selection of energy-absorbing device solutions can be divided into three stages, as shown in Figure 2.

Stage 1: Establish a systematic evaluation index system for the selection of bionic thin-wall structures.

The attributes are divided into several interval-value neutrosophic soft sets according to the different aspects of the description of the alternative, which can be considered as some approximate portrayal of the alternative, and these interval-value neutrosophic soft sets are combined by Definition 7 to obtain a more complete description (*F*, *E*) of the alternative. 

Stage 2: Obtain the weight vector of performance indicators by the IVNS-SOFT-ITARA method.

Subjective methods of extracting weights from DM could face many problems; for example, the accuracy of the evaluation decreases as the number of attributes increases, and these results are highly dependent on the method of assigning weights. This paper introduces a semi-objective method proposed in recent years that involves the innovative use of the IVNS-SOFT linguistic decision matrix to obtain the weights of attributes. 

Stage 3: Reduce the parameters and rank the alternative.

When the characteristics of a system described by IVNS-SOFT (*F*, *E*) can be fully represented by its core, *C*, then IVNS-SOFT (*F*, *C*) becomes the interval-valued neutrosophic soft-core set of IVNS-SOFT (*F*, *E*). According to the basic characteristics of multi-attribute decision problems and the parameter reduction algorithm, the interval-valued neutrosophic soft-core set is constructed (*F*, *C*). Then, the *Q_i_* value calculated by our proposed method determines the ranking of solutions, with higher *Q_i_* values indicating better solutions.

### 3.2. A Parameter Reduction Algorithm of Interval-Value Neutrosophic Soft Sets

Parameter reduction can remove unnecessarily existing parameters in the parameter set so that the remaining indispensable parameters in the parameter set maintain the same descriptive or decision-making ability as the original complete parameter set, e.g., normal parameter reduction [38], which is an adjustable parameter reduction approach for fuzzy soft sets based on the three-way decision [39]. Han et al. [40] transformed the soft-set parametric reduction problem into a 0–1 linear programming problem and developed a model for the pseudo-parametric reduction problem of soft sets, which was more efficient with a larger number of parameters. Ma and Qin [41] proposed a new parameter reduction algorithm based on the Euclidean distance named distance-based parameter reduction (DBPR). This method has higher applicability and less computational effort than the conventional fuzzy soft set parameter reduction method. 

This article presents the parameter reduction algorithm with interval-value neutrosophic soft sets as an extension of soft sets and fuzzy soft sets. The proposed Euclidean distance-based parameter reduction algorithm with interval-valued neutrosophic soft set is shown as follows in Algorithm 1:
**Algorithm 1**: Distance-based parameter reduction algorithm of interval-value neutrosophic soft sets**Input:** (*F*, *E*) (interval-valued neutrosophic soft set), *E* (parameter set), and threshold *λ* **Output:** (*E* − *A*) (an optimal choice considered parameter reduction in IVNS-SOFT). **BEGIN** 1. $\mathrm{Compute}\;\mathrm{the}\;\mathrm{weighted}\;\mathrm{matrix}\;{U=(u}_{ij}{)}_{m\times n}$, for 1 ≤ *i* ≤ *n*,1 ≤ *j* ≤ *m*. 2. Let *j* = 1. 3. $\mathrm{For}\;=1\;\mathrm{to}\;{(u}_{ij})$, **perform the following.** 4. Calculate distances for all pairs of *j* parameters. *j* = 1, 2, …, *m*. 5. Obtain the distance matrix *D* = (d*_kl_*)*_m×m_*, for 1 ≤ *k* ≤ *m*,1 ≤ *l* ≤ *m*. 6. **If** the distance between parameters *k* and *l* is less than the threshold *λ,*
**then perform the following.** 7. Consider parameters *k* and *l* to be similar, keep one parameter and place another set *A* of the reduction parameters. 8. Obtain the new interval-valued neutrosophic soft set (E−A) after parameter reduction. 9. **Return**
(E−A). **END**

When calculating the distance between two parameters, if the distance between two weighted parameters is less than the threshold *λ*, this paper considers that the similarity between these two parameters is high, and the weights of the parameters and the reasonableness of the reduction are taken into account at this point. Assuming that a parameter *e_p_* has high similarity with both the other two parameters, *e_a_* and *e_b_*, respectively, then we can choose to reduce *e_p_* instead of *e_a_* or *e_b_*. This parameter reduction method needs to establish a threshold value. Threshold *λ* is a measure of similarity for two attributes; threshold *λ* depends on the users. If the value *λ* is small, this means that users require a high degree of similarity in parameters.

If no such situation occurs, the one with the greater weight out of these two parameters can be kept. Parameter reduction through the consideration of weights is to prevent parameters with too large weights from having a large impact on the original criteria system after reduction. Then, reduced parameters can be placed in the approximate parameter set *A* while the other one is retained.

### 3.3. The ITARA Method with Interval-Value Neutrosophic Soft Sets

Barron et al. [42] stated that there is no consensus on which the subjective techniques method yields more accurate weights. In addition, decision makers may sometimes be unable to provide useful information for decision making. Combining objective and semi-objective methods to consider the determination of criteria allows the determination of objective weights involving the attitudes of decision makers, ultimately yielding more reliable results. The ITARA method does not require information from the decision makers but obtains the weights directly from the decision matrix data. The specific steps of the ITARA method are as follows.

Step 1: Construct the criteria system.

Step 2: Determine the normalized decision matrix${\;R=[\alpha }_{ij}{]}_{m\times n}$, where $\alpha_{ij}$ refers to the normalized aggregation of alternative *A_i_* under decision attributes. This paper proposed normalized aggregation through Equation (2).

(2) $$\alpha_{ij}=\frac{1}{6}{[(2+T}_{ij}^{L}-I_{ij}^{L}-F_{ij}^{L}{)+(2+T}_{ij}^{U}-I_{ij}^{U}-F_{ij}^{U})]/\sum_{i=1}^{m}\{\frac{1}{6}{[(2+T}_{ij}^{L}-I_{ij}^{L}-F_{ij}^{L}{)+(2+T}_{ij}^{U}-I_{ij}^{U}-F_{ij}^{U})]\}$$

Step 3: Rank normalized scores${\;\alpha }_{ij}\;$in ascending order, then name them as${\;\beta }_{ij}\;$in such a way that $\beta_{ij}\;\leq {\;\beta }_{i+1,\;j}$.

Step 4: Let $\gamma_{ij}{=\beta }_{i+1,\;j}{-\;\beta }_{ij}\left(i=1,\;2,\;\ldots ,\;m\;-\;1\right)$ be the ordered distances between adjacent${\;\beta }_{ij}\;$and${\;\beta }_{i+1,\;j}$.

Here, ${\;\gamma }_{ij}\;$presents the ordered distance between adjacent normalized aggregated assessments *β_i_*_+1,*j*_ and *β_ij_* under the decision attributes.

Step 5: As a matter of convenience, define the considerable difference between${\;\gamma }_{ij}\;$and *NIT_j_* as shown in Equaiton (S14), where *NIT* stands for the normalized indifference threshold. Determine a normalized indifference threshold for each attribute.

Step 6: Determine the weight vector of the attributes deduced from the *l_p_*-metric measurement by Equation (3).

(3) $$\omega_{j}{=v}_{j}\;/\overset{n}{\underset{j=1}{\sum }}v_{j}$$

${\mathrm{where}\;v}_{j}=(\sum_{i=1}^{m-1}\xi_{ij}^{p}{)}^{1/p},\;\forall j\in N$. In this study, *p* = 2 is selected.

### 3.4. The MABAC Method with Interval-Value Neutrosophic Soft Sets

For the evaluation of EADs, assume that the alternatives are as follows: *A_i_* = (*A*_1_, *A*_2_, …, *A_n_*); crashworthiness criteria: *CC* = {*CC*_1_, *CC*_2_, …, *CC_n_*}; and decision makers: *DM_k_* = {*DM*_1_, *DM*_2_, …, *DM_l_*}. Let *λ* = {*λ*_1_, *λ,* …, *λ_l_*} represent the weights of decision makers, meeting the requirement that 0 ≤ *λ_k_* ≤ 1 (*k* = 1, 2, …, *l*) and $\sum_{k=1}^{l}\lambda_{k}=1$. Each decision maker uses IVNS-SOFT to express the evaluation information. After that, expert decision matrices can be obtained $\tilde{E_{k}}=({\tilde{\mathrm{CC}}}_{ij}^{\left(k\right)}{)}_{m\times n}{=([T}_{ij}^{L(k)}{,\;T}_{ij}^{U(k)}{],\;[I}_{ij}^{L(k)}{,\;I}_{ij}^{U(k)}{],\;[F}_{ij}^{L(k)}{,\;F}_{ij}^{U(k)}])$*_m_*_×*n*_ (*k* = 1, 2, …, *l*). The detailed steps of the IVNS-SOFT-MABAC method are summarized as follows:

Step 1: Utilize the${\tilde{\;E}}_{k}\;$and aggregate decision matrices using the INSWG in Equation (S12): INSWG $x_{ij}{=\mathrm{INSWG}}_{\lambda }({\tilde{\mathrm{CC}}}_{ij}^{\left(1\right)},{\tilde{\mathrm{CC}}}_{ij}^{\left(2\right)},\;\ldots ,{\tilde{\;\mathrm{CC}}}_{ij}^{\left(l\right)})\;$to obtain $X=(x_{ij}{)}_{m\times n}$. Show the alternatives in the form of vectors *A_i_ =* (*x_i1_*, *x_i2_*, …, *x_in_*) through Equation (S16), where *x_ij_* is the value of the *i*-th alternative according to the *j*-th criterion (*i* = 1, 2, …, *m*; *j* = 1, 2, …, *n*).

Step 2: Obtain all criteria weight vectors using the ITARA method. Assign weights to the aggregation matrix *X* to obtain the weighted matrix *U.* The initial decision matrix *H* is obtained after parameter reduction using the improved DBPR method.

Step 3: Undertake the normalization of the elements from the initial matrix (*H*). Standardize this initial decision matrix. ${([T}_{F_{e_{j}}}^{L}{(h}_{i}{),\;T}_{F_{e_{j}}}^{U}{(h}_{i}{)],\;[I}_{F_{e_{j}}}^{L}{(h}_{i}{),\;I}_{F_{e_{j}}}^{U}{(h}_{i}{)],\;[F}_{F_{e_{j}}}^{L}{(h}_{i}{),\;F}_{F_{e_{j}}}^{U}{(h}_{i})])$ in ${([\tilde{T_{ij}^{L}},\;\tilde{T_{ij}^{U}}],\;[\tilde{I_{ij}^{L}},\;\tilde{I_{ij}^{U}}],\;[\tilde{F_{ij}^{L}},\tilde{{\;F}_{ij}^{U}}])}_{m\times n}$ by Equation (S17).

Step 4: Calculate the relative weights of the remaining attributes *C_j_* after parameter reduction.

Step 5: Compute the weighted matrix${\;Z=(z}_{ij}{)}_{m\times n}=$ $(\left[T_{ij}^{L},T_{ij}^{U}\right],\left[I_{ij}^{L},I_{ij}^{U}\right],\left[F_{ij}^{L},F_{ij}^{U}\right])\;$using Equations (S8) and (S18).

Step 6: Compute the border approximation area (BAA) matrix$\;{G=(g}_{j}{)}_{1\times n}$. The BAA for each attribute is obtained by Equation (4).

(4) $$g_{j}=\overset{m}{\underset{i=1}{\prod }}{{(z}_{ij})}^{1/m}=([\overset{m}{\underset{i=1}{\prod }}{{(T}_{ij}^{L})}^{1/m},\;\overset{m}{\underset{i=1}{\prod }}{{(T}_{ij}^{U})}^{1/m}],\;[1\;-\;\overset{m}{\underset{i=1}{\prod }}{{(1-I}_{ij}^{L})}^{1/m},\;1\;-\;\overset{m}{\underset{i=1}{\prod }}{{(1-I}_{ij}^{U})}^{1/m}],\;\;[1\;-\;\overset{m}{\underset{i=1}{\prod }}{{(1-F}_{ij}^{L})}^{1/m},\;1\;-\;\overset{m}{\underset{i=1}{\prod }}{{(1-F}_{ij}^{U})}^{1/m}])$$

where *z_ij_* represents the elements of the weighted matrix (Z).

After calculating the value *g_j_* for each criterion, a border approximation area matrix (*G*) is formed with the format 1 × *n*.

(5) $$G=\left[\begin{array}{cccc} g_{1} & g_{2} & \ldots & g_{n} \end{array}\right]$$

Step 7: Calculate the distance of the alternative from the border approximation area for the matrix elements (*D*) using Equation (S19).

Reckon the distance matrix${\;D=(d}_{ij}{)}_{m\times n}$ using Equation (S20). Size by the score function. If the scores of two IVNSNs are the same, the size is distinguished according to Definition 10.

**Figure 3:**
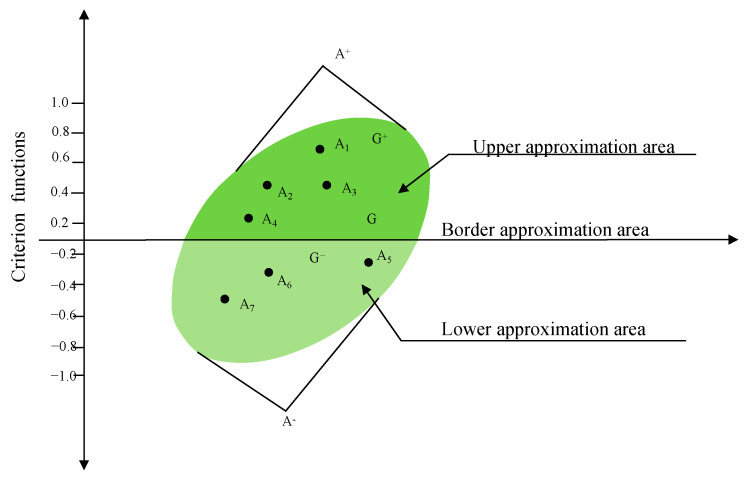
The upper (*G*^+^), lower (*G*^−^), and border (*G*) approximation areas.

The alternative *A_i_* could belong to the border approximation area (*G*), upper approximation area (*G*^+^), or lower approximation area (*G*^−^), as shown in Figure 3. Especially, the upper approximation area (G^+^) is the area which includes the ideal alternative (A^+^) while the lower approximation area (G^−^) is the area which includes the anti-ideal alternative (A^−^).

(6) $$A_{i}\in \left\{\begin{array}{ll} G^{+} & \mathrm{if}\;\;d_{ij}>0\\ G & \mathrm{if}\;d_{ij}=0\\ G^{-} & \mathrm{if}\;d_{ij}<0 \end{array}\right.$$

Step 8: The calculation from Equation (7) of the values for the alternatives is obtained as the sum of the distance of the alternatives from the border approximation areas (*d_i_*).

(7) $$Q_{i}=\sum_{j=1}^{n}d_{ij},i=1,\;\;2,\;\;\ldots ,\;\;m;j=1\;,2,\;\;\ldots ,\;\;n$$

Rank the alternatives by *Q_i_*. The optimal alternative is the one with the biggest value of *C_i_.*

## 4. Results and Discussion

The applicability and scientific validity of the discussed MCDM framework for bionic structure solutions are validated at different levels through a case study, comparative analysis, and sensitivity analysis.

### 4.1. Background

**Figure 4:**
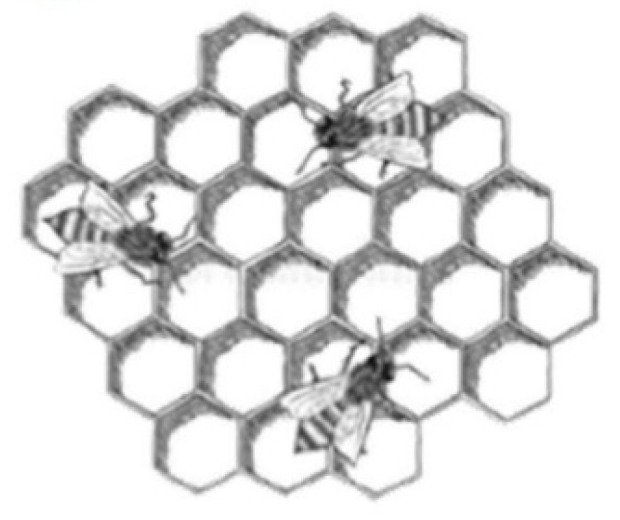
Bee honeycomb.

Bionic structures for high-speed trains are an area of research aimed at improving the safety and passenger comfort of high-speed trains by borrowing energy-absorbing mechanisms from biology; these structures have been the focus of research in the field of railroad passive safety [43]. At present, the research on structures can be divided into two categories as follows: the first is single-cell structures, and the second is multi-cell structures [44,45]. Compared with single-cell structures, multi-cell structures are important in engineering applications due to their good energy absorption capacity. This is because multi-cell structures produce more corner elements [46]. Among this type, thin-walled tubes are commonly used for energy absorption due to their lightweight and low manufacturing costs. In addition, high-speed trains have a dense arrangement of components, thus requiring structures to be as space-efficient as possible without compromising their energy-absorbing capacity. In nature, the most familiar and perfect example of a close arrangement is honeycomb, as shown in Figure 4.

In this section, the proposed decision method is validated against the engineering background of the honeycomb’s bionic thin-walled structure. 

### 4.2. Evaluation and Decision

**Figure 5:**
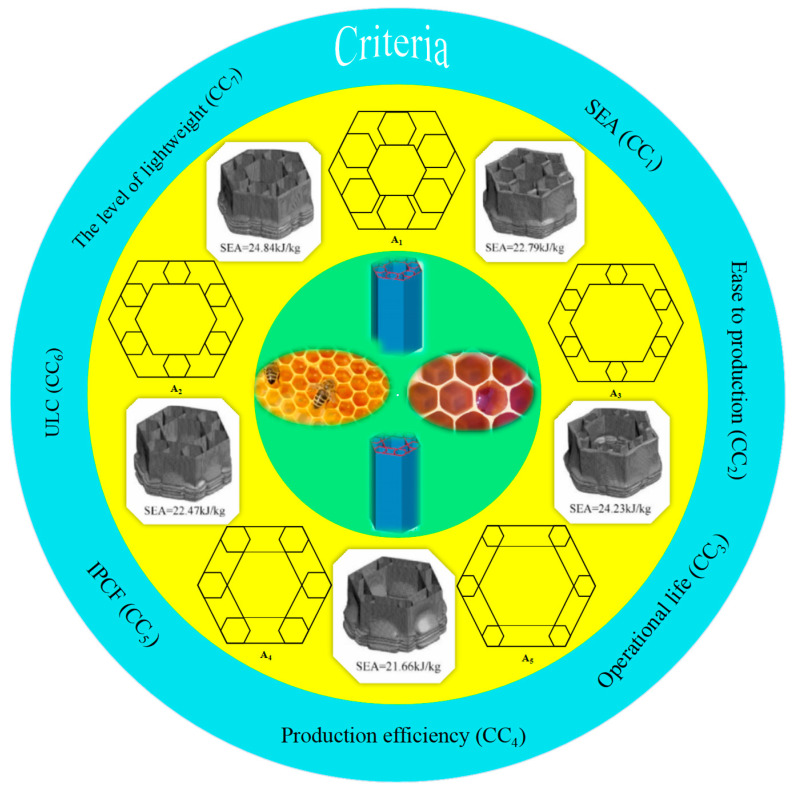
Five different structural alternatives.

In this case study, there were five honeycomb bionic thin-walled structures of the same material but different configurations; these included both wall-to-wall (WTW) and corner-to-corner (CTC) configurations, five structures were obtained for SEA by experiments, in addition this case study includes seven criteria, as shown in Figure 5. 

The height of the bionic thin-walled structure is 150 mm, the wall length of the structural outline is 45 mm, and the wall thickness is 1 mm. Crashworthiness criteria are necessary to evaluate crashworthiness performance. Usually, different criteria are used for different engineering requirements [47,48]. From the perspective of energy-absorbing safety, the structure should absorb more energy and have higher energy-absorbing efficiency, and at the same time, reduce the level of peak force during a collision to prevent overload impacting the safety of passengers. From the perspective of energy-absorbing stability, a force curve without significant fluctuation is an ideal structure. Therefore, in this study, SEA, IPCF, and the undulation of load-carrying capacity (ULC) are specifically considered to study the energy absorption of structure. Based on the current global concept of sustainable development, the important sustainability properties of structures for high-speed trains should also be considered. In this paper, the following criteria were used to evaluate the crashworthiness of thin-walled tubes, i.e., SEA (CC_1_), ease of production (CC_2_), operational life (CC_3_), production efficiency (CC_4_), IPCF (CC_5_), ULC (CC_6_), and their lightweight level (CC_7_), where CC_1_-CC_4_ represent the benefit type indicators and CC_5_-CC_7_ represent the cost type indicators. An evaluation of linguistic terms is shown in Table 1. 

The evaluation results obtained by three experts (namely E_1_, E_2,_ and E_3_) from different industries, e.g., university academics and engineering designers, found λ= (1/3, 1/3, 1/3)^T^ to be the weights vector. The expert decision matrices are shown in Tables S1–S3. 

Each expert evaluated each alternative against each criterion. There were five linguistic variables for the language terms selected for this study. Each language level contained the following elements: (1) the extent to which the expert considered the criteria to be important; (2) the extent to which the expert considered the criteria unimportant; and (3) the extent to which the expert was uncertain about the criteria. The IVNS-SOFT can describe all three types of information.

Step 1: Aggregate expert decision matrix [$\tilde{E_{k}}]\;$(*k* = 1, 2, …, *l*). In this case study, the scores of subjective criteria in the expert decision matrix were expressed through expert evaluation. The scores of objective criteria were obtained from the numerical analysis results of structures and expressed through the IVNS-SOFT. It is worth noting that in IVNS-SOFT, the range of values is required to be 0–1, so it is necessary to normalize the values of the objective indicators using Equation (8). The integrated decision matrix [*X*] is obtained through Equation (S12) and shown in Table S4.

(8) $$x_{ij}=\left\{\begin{array}{c} \frac{r_{\max j}-r_{ij}}{r_{\max j}-r_{\min j}}\;\\ \frac{r_{ij}-r_{\min j}}{r_{\max j}-r_{\min j}} \end{array}\right.$$

For the cost criteria, $x_{ij}=\frac{r_{\max j}-r_{ij}}{r_{\max j}-r_{\min j}}$, and for the benefit criteria, $x_{ij}=\frac{r_{ij}-r_{\min j}}{r_{\max j}-r_{\min j}}$. 

Here, $r_{\max j}$ represents the maximum value of the numerical analysis results and $r_{\min j}$ represents the minimum value of the numerical analysis results. 

Step 2: The calculation process of the seven criteria weights and their corresponding normalized weights using Equation (3) are shown in Table S5.

Step 3: The combined weighting matrix is obtained through Equation (S6) and shown in Table S6.

Step 4: We calculated the Euclidean distance between each of the seven parameters using Equation (S13), and the Euclidean distance is shown in Table S7.

Step 5: Here, λ = 0.1000, and when scanning the Euclidean distance matrix, it was found that the Euclidean distance of CC_3_ and CC_6_ was smaller than the threshold value, and according to the parameter reduction rule, CC_3_ was chosen to be removed in this paper. In fact, in structures, CC_6_ is indeed similar to CC_3_. A smaller CC_6_ indicates a better energy absorption efficiency; the load-carrying fluctuation is also small, and the operational life is longer. In addition, the Euclidean distances of CC_2_ and CC_4_ are also smaller than the threshold, and CC_4_ with smaller weights was selected for reduction in this paper.

Step 6: Obtain the IVNS-SOFT decision matrix after parameter reduction. The multi-attribute decision problem is described in terms of this interval-valued neutrosophic soft set.

Step 7: Normalize the initial matrix using Equation (S17), as shown in Table S8.

Step 8: The relative weights of the reduced parameters are calculated, as shown in Table 2.

Step 9: Compute the weighted normalized matrix *Z* = (*z_ij_*)*_m_*_×*n*_ using Equation (S18), as shown in Table S9.

Step 10: The border approximation area (BAA) matrix is calculated *G* = (*g_j_*)_1×*n*_ using Equation (4) as shown in Table S10.

Step 11: The distance matrix *D* = (*d_ij_*)*_m_*_×*n*_ using Equation (S20) is calculated, as shown in Table 3. 

Step 12: The *Q_i_* scores using Equation (7) for energy-absorbing devices are as follows: *Q*_1_ = 1.7292, *Q*_2_ = 1.9593, *Q*_3_ = 0.9961, *Q*_4_ = 0.6806, *Q*_5_ = −0.1343. The final sorting of the individual programs can then be obtained, i.e., A_2_ > A_1_ > A_3_ > A_4_ > A_5_, where A_2_ is the best structure solution. 

### 4.3. Comparative Studies with Other Traditional Methods

In this section, a comparative experiment of different MCDM methods is presented to prove the scientific validity and rationality of the method proposed in this paper. Four traditional methods, including SVNS-VIKOR(VlseKriterijumska Optimizacija I Kompromisno Resenje) [49], IVNS-VIKOR [50], SVNS-MABAC [51], and similarity measures between IVNSs are included [37]. The sort results generated by these four methods are presented in Table 4.

It can be found that the sort results of the IVNS-SOFT-MABAC method are completely consistent with the SVNS-MABAC method, with similarity measures between the IVNSs method. Therefore, the availability and effectiveness of the proposed IVNS-SOFT-MABAC method can be validated. The SVNS-VIKOR method and the IVNS-VIKOR method are a little different in the sorting of A_3_ and A_4_. It infers that the gap between these two schemes is small. The IVNS-VIKOR and the SVNS-VIKOR cannot be distinguished any better. In addition, the VIKOR method differs from the MABAC method in the selection of an optimal solution, which indicates that the VIKOR method does not accurately distinguish between optimal and suboptimal solutions.

SVNS uses a single exact value to represent each affiliation grade, but sometimes, the affiliation grade is uncertain and difficult to define with a clear value. The IVNS-SOFT fuzzy theory uses interval values to represent three affiliation grades of fuzzy sets, which consider the fuzziness of the affiliation grade, and the uncertainty is explicitly quantified, which can deal with the uncertain and inconsistent information that is prevalent in problems. Whereas the VIKOR method usually provides a compromise and alternatives, the MABAC method is more reliable for optimal choices. Compared to the existing literature, the MABAC method is based on the distance of each alternative to the border approximation matrix for each criterion, whose valuable advantage is that it takes into account the uncertainty of the decision maker and the ambiguity of the decision environment, whose solution is stable, and this leads to more accurate and effective decision-making, which stands out from the crowd of evaluation methods. The above analysis can prove the significant advantages of the method and provide a new decision-making method for decision makers.

### 4.4. Sensitivity Analysis 

**Figure 6:**
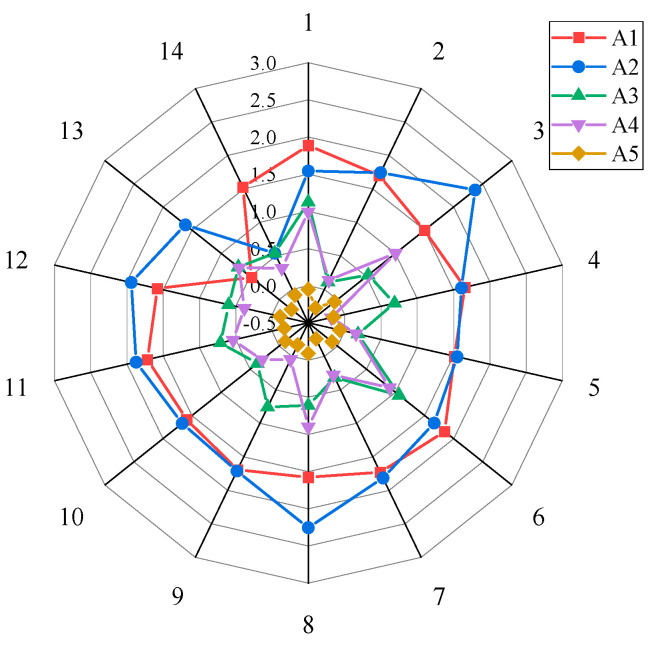
Results of sensitivity analysis for 14 experiments.

In this section, a sensitivity analysis is conducted to explore the effect of changes in weights on the ranking of SCT schemes. In the sensitivity analysis, 14 experiments were designed to record the final rank, and the *Q_i_* values are shown in Table 5 and Figure 6. In the first 10 experiments, the weights of each major criterion were set to the highest, and the weights of the corresponding other indicators were set the same. In experiment 11, the weights of each criterion were set to be the same. In experiment 12, the weights of benefit indicators were set to 0.050, and the weights of cost indicators were set to 0.300. In experiment 13, the weights of benefit indicators were set to 0.500, and the weights of cost indicators were set to 0.000. In experiment 14, the weights of benefit indicators were set to 0.000, and the weights of cost indicators were set to 0.333.

As seen in Table 5 and Figure 6, some conclusions can be summed up as follows: (1) the final ranking of each structure alternative varies greatly with the change in each indicator’s weight; therefore, the construction of a reasonable multi-criteria weighting system based on the requirements of the engineering context has a great impact on the results. (2) When these criteria, i.e., CC_1_ and CC_6_, reach a large proportion, respectively, A_1_ has the highest result score; similarly, when CC_2_, CC_5_, and CC_7_ reach a large proportion, A_2_ has the highest score. With the change in the criteria system, the results change significantly. The variation between the sort results shows that the standard weights play a vital role in the evaluation of energy-absorbing devices. Determining accurate weights helps to provide better options for selecting bionic thin-walled structures.

## 5. Conclusions

In this paper, a parameter reduction-based indifference threshold-based attribute ratio analysis method under IVNS-SOFT is proposed to obtain the weight vector of the evaluation indicator system for the selection of bionic thin-wall structures. An IVNS-SOFT-based MABAC method is proposed to obtain the optimal alternative. Subsequently, an application of five bio-inspired thin-wall structures was applied to demonstrate that this proposed method is valid and practical. Comparative analysis, sensitivity analysis, and discussion are conducted in this research. Based on the results obtained in this study, several conclusions are summarized as follows:

(1) Different design philosophies of bionic thin-wall structures lead to different performance and application scenarios.
(2) Indicators of SEA, ease of production, IPCF, ULC, and lightweight levels greatly affect the process of bionic thin-wall structure selection, with weights of 0.2596, 0.0960, 0.2929, 0.3336 and 0.0179, respectively.
(3) By comparing this with the four existing methods, it was found that the proposed method is reasonable and feasible to select optimal bionic thin-wall structures.
(4) Through sensitivity analysis, the results found that out of 14 experiments, alternative A2 had the highest score in 10 experiments. Hence, the final result is reliable. In addition, the rank of each alternative is relatively sensitive to the criteria weights.

In future studies, several directions may be investigated in this research as follows: (1) constructing a different criteria system for the structure to demonstrate the applicability of the method under different conditions; (2) investigating the hybrid decision-making method combined with the weight model to solve the problem of objective and subjective weight information.

## Figures and Tables

**Table 1:** Linguistic terms for IVNS-SOFT.

Linguistic Variable	Interval-Valued Neutrosophic Soft Number
Unimportant (UI)	<[0.1, 0.2], [0.4, 0.5], [0.6, 0.7]>
Ordinary level of importance (OI)	<[0.2, 0.4], [0.5, 0.6], [0.4, 0.5]>
Important (IM)	<[0.4, 0.6], [0.4, 0.5], [0.3, 0.4]>
Very important (VI)	<[0.6, 0.8], [0.3, 0.4], [0.2, 0.3]>
Absolutely important (AI)	<[0.7, 0.9], [0.2, 0.3], [0.1, 0.2]>

**Table 2:** The relative weights *CC_j_*.

	CC_1_	CC_2_	CC_5_	CC_6_	CC_7_
Weight	0.2596	0.0960	0.2929	0.3336	0.0179

**Table 3:** The distance matrix.

Alt.	CC_1_	CC_2_	CC_5_	CC_6_	CC_7_
A_1_	0.1523	0.0599	1.4142	0.1021	0.0006
A_2_	1.4142	0.0599	0.4472	0.0378	0.0003
A_3_	0.4931	−0.0275	0.1407	0.3904	−0.0005
A_4_	0.4227	−0.0050	0.4722	−0.2096	−0.0003
A_5_	0.0000	−0.0576	0.0000	−0.0761	−0.0005

**Table 4:** The results of different methods.

Methods	Results
SVNS-VIKOR	A_1_ > A_2_ > A_4_ > A_3_ > A_5_
IVNS-VIKOR	A_1_ > A_2_ > A_3_ > A_4_ > A_5_
SVNS-MABAC	A_2_ > A_1_ > A_3_ > A_4_ > A_5_
Similarity measures between IVNSs	A_2_ > A_1_ > A_3_ > A_4_ > A_5_
The proposed method	A_2_ > A_1_ > A_3_ > A_4_ > A_5_

**Table 5:** Results of sensitivity analysis.

No.	Weights	*Q_i_* Value					Rank
		A_1_	A_2_	A_3_	A_4_	A_5_	
1	*ω*_CC1_ = 0.800, *ω*_CC2, CC5–7_ = 0.050	1.8864	1.5430	1.1276	0.9949	−0.0479	A_1_ > A_2_ > A_3_ > A_4_ > A_5_
2	*ω*_CC2_ = 0.800,*ω*_CC1, CC5–7_ = 0.050	1.6988	1.7435	0.1108	0.1395	−0.2756	A_2_ > A_1_ > A_4_ > A_3_ > A_5_
3	*ω*_CC5_ = 0.800, *ω*_CC1–2, CC6–7_ = 0.050	1.4983	2.3677	0.5298	1.0011	−0.0479	A_2_ > A_1_ > A_4_ > A_3_ > A_5_
4	*ω*_CC6_ = 0.800, *ω*_CC1–2, CC5, CC7_ = 0.050	1.6570	1.6122	0.6896	−0.1818	−0.1517	A_1_ > A_2_ > A_3_ > A_5_ > A_4_
5	*ω*_CC7_ = 0.800, *ω*_CC1–2, CC5–6_ = 0.050	1.5173	1.5521	0.1853	0.1567	−0.0654	A_2_ > A_1_ > A_3_ > A_4_ > A_5_
6	*ω*_CC1_ = 0.600, *ω*_CC2, CC5–7_ = 0.100	1.8433	1.6623	1.0620	0.8970	−0.0914	A_1_ > A_2_ > A_3_ > A_4_ > A_5_
7	*ω*_CC2_ = 0.600,*ω*_CC1, CC5–7_ = 0.100	1.7298	1.8155	0.3173	0.2791	−0.2617	A_2_ > A_1_ > A_3_ > A_4_ > A_5_
8	*ω*_CC5_ = 0.600, *ω*_CC1–2, CC6–7_ = 0.100	1.5766	2.2553	0.6117	0.9020	−0.0914	A_2_ > A_1_ > A_4_ > A_3_ > A_5_
9	*ω*_CC6_ = 0.600, *ω*_CC1–2, CC5, CC7_ = 0.100	1.6927	1.7112	0.7573	0.0459	−0.1694	A_2_ > A_1_ > A_3_ > A_4_ > A_5_
10	*ω*_CC7_ = 0.600, *ω*_CC1–2, CC5–6_ = 0.100	1.5899	1.6686	0.3759	0.2924	−0.1036	A_2_ > A_1_ > A_3_ > A_4_ > A_5_
11	*ω*_CC1–2, CC5–7_ = 0.200	1.7177	1.8752	0.7124	0.5373	−0.1668	A_2_ > A_1_ > A_3_ > A_4_ > A_5_
12	*ω*_CC1–2_ = 0.050, *ω*_CC5–7_ = 0.300	1.5810	1.9419	0.5976	0.3835	−0.1103	A_2_ > A_1_ > A_3_ > A_4_ > A_5_
13	*ω*_CC1–2_ = 0.500, *ω*_CC5–7_ = 0.000	0.4782	1.6137	0.7027	0.6882	−0.2090	A_2_ > A_3_ > A_4_ > A_1_ > A_5_
14	*ω*_CC1–2_ = 0.000, *ω*_CC5–7_ = 0.333	1.5262	0.5387	0.5397	0.3186	−0.0852	A_1_ > A_3_ > A_2_ > A_4_ > A_5_

## Data Availability

The datasets used and/or analyzed during the current study are available from the corresponding author on reasonable request. All data generated or analyzed during this study are included in this article.

## Supplementary Materials

The following supporting information can be downloaded at: https://www.mdpi.com/article/10.3390/biomimetics9040208/s1, Table S1. The IVNS-SOFTs decision matrix by E1. Table S2. The IVNS-SOFTs decision matrix by E2. Table S3. The IVNS-SOFTs decision matrix by E3. Table S4. Integrated decision matrix. Table S5. ITARA calculation process and the criteria weight. Table S6. Weighted decision matrix. Table S7. Distance weighted matrix. Table S8. The normalization initial matrix. Table S9. The weighted matrix. Table S10. The border approximation area (BAA) matrix. Supplementary Material B: Equation (S1)–(S20).
