# Supplementary material for: A Parameter Reduction-Based Decision-Making Method with Interval-Valued Neutrosophic Soft Sets for the Selection of Bionic Thin-Wall Structures
Source: Biomimetics (Basel). 2024 Mar 29;9(4):208. doi: 10.3390/biomimetics9040208 (PMC11048664; doi:10.3390/biomimetics9040208)
Supplement: Supplementary file 1 [file biomimetics-09-00208-s001.zip › biomimetics-2897358-supplementary.pdf]

biomimetics

Supplementary Materials

A Parameter Reduction-Based Decision-Making Method with Interval-Valued Neutrosophic Soft Sets for the Selection of Bionic Thin-Wall Structures

Honghao Zhang [1,2], Lingyu Wang [1], Danqi Wang [3,*], Zhongwei Huang [1,*], Dongtao Yu [1] and Yong Peng [4]

1 Key Laboratory of High Efficiency and Clean Mechanical Manufacture (Ministry of Education), School of Mechanical Engineering, Shandong University, Jinan 250061, China; honghao_zhang@sdu.edu.cn (H.Z.); 202214329@mail.sdu.edu.cn (L.W.); dongtaoyu2022@mail.sdu.edu.cn (D.Y.)
2 Key Laboratory of Transportation Industry for Transport Vehicle Detection, Diagnosis and Maintenance Technology, Jinan 250061, China
3 College of Automotive and Mechanical Engineering, Changsha University of Science and Technology, Changsha 410114, China
4 Key Laboratory of Traffic Safety on Track of Ministry of Education, School of Traffic and Transportation Engineering, Central South University, Changsha 410083, China; yong_peng@csu.edu.cn
* Correspondence: danqi_wang@csust.edu.cn (D.W.); huangzhongwei@mail.sdu.edu.cn (Z.H.)

Citation: Zhang, H.; Wang, L.; Wang, D.; Huang, Z.; Yu, D.; Peng, Y. A Parameter Reduction-Based Decision-Making Method with Interval-Valued Neutrosophic Soft Sets for the Selection of Bionic Thin-Wall Structures. *Biomimetics* **2024**, *9*, 208. https://doi.org/10.3390/biomimetics9040208

Academic Editor: Yongmei Zheng

Received: 18 February 2024
Revised: 19 March 2024
Accepted: 27 March 2024
Published: 29 March 2024

Copyright: © 2024 by the authors. Licensee MDPI, Basel, Switzerland. This article is an open access article distributed under the terms and conditions of the Creative Commons Attribution (CC BY) license (https://creativecommons.org/licenses/by/4.0/).

Supplementary Material A

Table S1. The IVNS-SOFTs decision matrix by E_1.

Alt.	CC_1	CC_2	CC_3	CC_4	CC_5	CC_6	CC_7
A_1	(0.3553, 0.3553, 0.0000, 0.0000, 0.0000, 0.0000)	VI	OI	IM	(0.0000, 0.0000, 0.0000, 0.0000, 0.0000, 0.0000)	OI	(0.7180, 0.7180, 0.0000, 0.0000, 0.0000, 0.0000)
A_2	(1.0000, 1.0000, 0.0000, 0.0000, 0.0000, 0.0000)	VI	OI	VI	(0.2731, 0.2731, 0.0000, 0.0000, 0.0000, 0.0000)	OI	(0.7270, 0.7270, 0.0000, 0.0000, 0.0000, 0.0000)
A_3	(0.8082, 0.8082, 0.0000, 0.0000, 0.0000, 0.0000)	IM	UI	VI	(0.6992, 0.6992, 0.0000, 0.0000, 0.0000, 0.0000)	UI	(0.7510, 0.7510, 0.0000, 0.0000, 0.0000, 0.0000)
A_4	(0.7453, 0.7453, 0.0000, 0.0000, 0.0000, 0.0000)	VI	AI	IM	(0.2497, 0.2497, 0.0000, 0.0000, 0.0000, 0.0000)	VI	(0.7270, 0.7270, 0.0000, 0.0000, 0.0000, 0.0000)
A_5	(0.0000, 0.0000, 0.0000, 0.0000, 0.0000, 0.0000)	OI	VI	VI	(1.0000, 1.0000, 0.0000, 0.0000, 0.0000, 0.0000)	OI	(0.7510, 0.7510, 0.0000, 0.0000, 0.0000, 0.0000)

Table S2. The IVNS-SOFTs decision matrix by E_2.

Alt.	CC_1	CC_2	CC_3	CC_4	CC_5	CC_6	CC_7
A_1	(0.3553, 0.3553, 0.0000, 0.0000, 0.0000, 0.0000)	VI	IM	VI	(0.0000, 0.0000, 0.0000, 0.0000, 0.0000, 0.0000)	OI	(0.7180, 0.7180, 0.0000, 0.0000, 0.0000, 0.0000)
A_2	(1.0000, 1.0000, 0.0000, 0.0000, 0.0000, 0.0000)	VI	OI	VI	(0.2731, 0.2731, 0.0000, 0.0000, 0.0000, 0.0000)	IM	(0.7270, 0.7270, 0.0000, 0.0000, 0.0000, 0.0000)
A_3	(0.8082, 0.8082, 0.0000, 0.0000, 0.0000, 0.0000)	IM	OI	IM	(0.6992, 0.6992, 0.0000, 0.0000, 0.0000, 0.0000)	UI	(0.7510, 0.7510, 0.0000, 0.0000, 0.0000, 0.0000)
A_4	(0.7453, 0.7453, 0.0000, 0.0000, 0.0000, 0.0000)	IM	OI	OI	(0.2497, 0.2497, 0.0000, 0.0000, 0.0000, 0.0000)	VI	(0.7270, 0.7270, 0.0000, 0.0000, 0.0000, 0.0000)
A_5	(0.0000, 0.0000, 0.0000, 0.0000, 0.0000, 0.0000)	VI	VI	OI	(1.0000, 1.0000, 0.0000, 0.0000, 0.0000, 0.0000)	VI	(0.7510, 0.7510, 0.0000, 0.0000, 0.0000, 0.0000)

Table S3. The IVNS-SOFTs decision matrix by E_3.

Alt.	CC_1	CC_2	CC_3	CC_4	CC_5	CC_6	CC_7
A_1	(0.3553, 0.3553, 0.0000, 0.0000, 0.0000, 0.0000)	VI	OI	VI	(0.0000, 0.0000, 0.0000, 0.0000, 0.0000, 0.0000)	IM	(0.7180, 0.7180, 0.0000, 0.0000, 0.0000, 0.0000)
A_2	(1.0000, 1.0000, 0.0000, 0.0000, 0.0000, 0.0000)	VI	IM	IM	(0.2731, 0.2731, 0.0000, 0.0000, 0.0000, 0.0000)	IM	(0.7270, 0.7270, 0.0000, 0.0000, 0.0000, 0.0000)
A_3	(0.8082, 0.8082, 0.0000, 0.0000, 0.0000, 0.0000)	IM	UI	IM	(0.6992, 0.6992, 0.0000, 0.0000, 0.0000, 0.0000)	UI	(0.7510, 0.7510, 0.0000, 0.0000, 0.0000, 0.0000)
A_4	(0.7453, 0.7453, 0.0000, 0.0000, 0.0000, 0.0000)	IM	VI	IM	(0.2497, 0.2497, 0.0000, 0.0000, 0.0000, 0.0000)	VI	(0.7270, 0.7270, 0.0000, 0.0000, 0.0000, 0.0000)
A_5	(0.0000, 0.0000, 0.0000, 0.0000, 0.0000, 0.0000)	OI	VI	VI	(1.0000, 1.0000, 0.0000, 0.0000, 0.0000, 0.0000)	VI	(0.7510, 0.7510, 0.0000, 0.0000, 0.0000, 0.0000)

Table S4. Integrated decision matrix.

Alt.	CC_1	CC_2	CC_3	CC_4	CC_5	CC_6	CC_7
A_1	(0.3553, 0.3553, 0.0000, 0.0000, 0.0000, 0.0000)	(0.6000, 0.8000, 0.3000, 0.4000, 0.2000, 0.3000)	(0.2520, 0.4579, 0.4687, 0.5691, 0.3684, 0.4687)	(0.5241, 0.7268, 0.3351, 0.4354, 0.2348, 0.3351)	(0.0000, 0.0000, 0.0000, 0.0000, 0.0000, 0.0000)	(0.2520, 0.4579, 0.4687, 0.5691, 0.3684, 0.4687)	(0.7180, 0.7180, 0.0000, 0.0000, 0.0000, 0.0000)
A_2	(1.0000, 1.0000, 0.0000, 0.0000, 0.0000, 0.0000)	(0.6000, 0.8000, 0.3000, 0.4000, 0.2000, 0.3000)	(0.2520, 0.4579, 0.4687, 0.5691, 0.3684, 0.4687)	(0.5241, 0.7268, 0.3351, 0.4354, 0.2348, 0.3351)	(0.2731, 0.2731, 0.0000, 0.0000, 0.0000, 0.0000)	(0.3175, 0.5241, 0.4354, 0.5358, 0.3351, 0.4354)	(0.7270, 0.7270, 0.0000, 0.0000, 0.0000, 0.0000)

A3	(0.8082, 0.8082, 0.0000, 0.0000, 0.0000, 0.0000)	(0.4000, 0.6000, 0.4000, 0.5000, 0.3000, 0.4000)	(0.1260, 0.2520, 0.4354, 0.5358, 0.5421, 0.6443)	(0.4579, 0.6604, 0.3684, 0.4687, 0.2681, 0.3684)	(0.6992, 0.6992, 0.0000, 0.0000, 0.0000, 0.0000)	(0.1000, 0.2000, 0.4000, 0.5000, 0.6000, 0.7000)	(0.7510, 0.7510, 0.0000, 0.0000, 0.0000, 0.0000)
A4	(0.7453, 0.7453, 0.0000, 0.0000, 0.0000, 0.0000)	(0.4579, 0.6604, 0.3684, 0.4687, 0.2681, 0.3684)	(0.4380, 0.6604, 0.3458, 0.4482, 0.2440, 0.3458)	(0.3175, 0.5241, 0.4354, 0.5358, 0.3351, 0.4354)	(0.2497, 0.2497, 0.0000, 0.0000, 0.0000, 0.0000)	(0.6000, 0.8000, 0.3000, 0.4000, 0.2000, 0.3000)	(0.7270, 0.7270, 0.0000, 0.0000, 0.0000, 0.0000)
A5	(0.0000, 0.0000, 0.0000, 0.0000, 0.0000, 0.0000)	(0.2884, 0.5040, 0.4407, 0.5421, 0.3396, 0.4407)	(0.6000, 0.8000, 0.3000, 0.4000, 0.2000, 0.3000)	(0.4160, 0.6350, 0.3743, 0.4759, 0.2732, 0.3743)	(1.0000, 1.0000, 0.0000, 0.0000, 0.0000, 0.0000)	(0.4160, 0.6350, 0.3743, 0.4759, 0.2732, 0.3743)	(0.7510, 0.7510, 0.0000, 0.0000, 0.0000, 0.0000)

Table S5. ITARA calculation process and the criteria weight.

	CC1	CC2	CC3	CC4	CC5	CC6	CC7
β_{ij}	0.1549	0.1640	0.1405	0.1711	0.1636	0.1330	0.1988
	0.1825	0.1840	0.1794	0.1961	0.1841	0.1796	0.1994
	0.2127	0.1973	0.1794	0.2011	0.1860	0.1963	0.1994
	0.2175	0.2273	0.2350	0.2158	0.2208	0.2251	0.2012
	0.2324	0.2273	0.2657	0.2158	0.2455	0.2660	0.2012
NIT_j	0.0100	0.0200	0.0300	0.0200	0.0100	0.0200	0.0000
γ_{ij}	0.0275	0.0201	0.0389	0.0250	0.0204	0.0466	0.0007
	0.0302	0.0132	0.0000	0.0050	0.0019	0.0168	0.0000
	0.0049	0.0301	0.0556	0.0147	0.0349	0.0287	0.0018
	0.0149	0.0000	0.0307	0.0000	0.0246	0.0410	0.0000
ξ_{ij}	0.0175	0.0001	0.0089	0.0050	0.0104	0.0266	0.0007
	0.0202	0.0000	0.0000	0.0000	0.0000	0.0000	0.0000
	0.0000	0.0101	0.0256	0.0000	0.0249	0.0087	0.0018
	0.0049	0.0000	0.0007	0.0000	0.0146	0.0210	0.0000
v_j	0.0272	0.0101	0.0272	0.0050	0.0307	0.0349	0.0019
ω_j	0.1986	0.0735	0.1983	0.0367	0.2240	0.2552	0.0137

Table S6. Weighted decision matrix.

**Alt.**	**CC1**	**CC2**	**CC3**	**CC4**	**CC5**	**CC6**	**CC7**
A1	(0.0835, 0.0835, 0.0000, 0.0000, 0.0000, 0.0000)	(0.0651, 0.1115, 0.9154, 0.9349, 0.8885, 0.9154)	(0.0560, 0.1144, 0.8604, 0.8942, 0.8203, 0.8604)	(0.0269, 0.0465, 0.9606, 0.9699, 0.9482, 0.9606)	(0.0000, 0.0000, 0.0000, 0.0000, 0.0000, 0.0000)	(0.0714, 0.1446, 0.8242, 0.8660, 0.7750, 0.8242)	(0.0172, 0.0172, 0.0000, 0.0000, 0.0000, 0.0000)
A2	(1.0000, 1.0000, 0.0000, 0.0000, 0.0000, 0.0000)	(0.0651, 0.1115, 0.9154, 0.9349, 0.8885, 0.9154)	(0.0560, 0.1144, 0.8604, 0.8942, 0.8203, 0.8604)	(0.0269, 0.0465, 0.9606, 0.9699, 0.9482, 0.9606)	(0.0690, 0.0690, 0.0000, 0.0000, 0.0000, 0.0000)	(0.0929, 0.1726, 0.8088, 0.8528, 0.7565, 0.8088)	(0.0176, 0.0176, 0.0000, 0.0000, 0.0000, 0.0000)
A3	(0.2796, 0.2796, 0.0000, 0.0000, 0.0000, 0.0000)	(0.0368, 0.0651, 0.9349, 0.9504, 0.9154, 0.9349)	(0.0264, 0.0560, 0.8480, 0.8836, 0.8856, 0.9165)	(0.0222, 0.0389, 0.9640, 0.9726, 0.9528, 0.9640)	(0.2359, 0.2359, 0.0000, 0.0000, 0.0000, 0.0000)	(0.0265, 0.0553, 0.7915, 0.8379, 0.8778, 0.9130)	(0.0189, 0.0189, 0.0000, 0.0000, 0.0000, 0.0000)
A4	(0.2739, 0.2739, 0.0000, 0.0000, 0.0000, 0.0000)	(0.0440, 0.0763, 0.9293, 0.9458, 0.9078, 0.9293)	(0.1080, 0.1928, 0.8101, 0.8529, 0.7560, 0.8101)	(0.0139, 0.0269, 0.9699, 0.9774, 0.9606, 0.9699)	(0.0623, 0.0623, 0.0000, 0.0000, 0.0000, 0.0000)	(0.2085, 0.3368, 0.7355, 0.7915, 0.6632, 0.7355)	(0.0176, 0.0176, 0.0000, 0.0000, 0.0000, 0.0000)
A5	(0.0000, 0.0000, 0.0000, 0.0000, 0.0000, 0.0000)	(0.0247, 0.0502, 0.9416, 0.9560, 0.9237, 0.9416)	(0.1662, 0.2733, 0.7876, 0.8338, 0.7267, 0.7876)	(0.0196, 0.0363, 0.9646, 0.9731, 0.9535, 0.9646)	(1.0000, 1.0000, 0.0000, 0.0000, 0.0000, 0.0000)	(0.1282, 0.2267, 0.7782, 0.8274, 0.7181, 0.7782)	(0.0189, 0.0189, 0.0000, 0.0000, 0.0000, 0.0000)

Table S7. Distance weighted matrix.

D	CC_1	CC_2	CC_3	CC_4	CC_5	CC_6	CC_7
CC1	0	1.6193	1.4823	1.6875	0.5265	1.4109	0.3650
CC2	1.6193	0	0.1719	0.0767	1.6208	0.2498	1.5448
CC3	1.4823	0.1719	0	0.2409	1.4622	0.0960	1.4060
CC4	1.6875	0.0767	0.2409	0	1.6829	0.3208	1.6056
CC5	0.5265	1.6208	1.4622	1.6829	0	1.4078	0.3091
CC6	1.4109	0.2498	0.0960	0.3208	1.4078	0	1.3452
CC7	0.3650	1.5448	1.4060	1.6056	0.3091	1.3452	0

Table S8. The normalization initial matrix.

Alt.	CC_1	CC_2	CC_5	CC_6	CC_7
A_1	(0.3553, 0.3553, 0.0000, 0.0000, 0.0000, 0.0000)	(0.6000, 0.8000, 0.3000, 0.4000, 0.2000, 0.3000)	(0.0000, 0.0000, 1.0000, 1.0000, 0.0000, 0.0000)	(0.3684, 0.4687, 0.4309, 0.5313, 0.2520, 0.4579)	(0.0000, 0.0000, 1.0000, 1.0000, 0.7180, 0.7180)
A_2	(1.0000, 1.0000, 0.0000, 0.0000, 0.0000, 0.0000)	(0.6000, 0.8000, 0.3000, 0.4000, 0.2000, 0.3000)	(0.0000, 0.0000, 1.0000, 1.0000, 0.2731, 0.2731)	(0.3351, 0.4354, 0.4642, 0.5646, 0.3175, 0.5241)	(0.0000, 0.00000, 1.0000, 1.0000, 0.7270, 0.7270)
A_3	(0.8082, 0.8082, 0.0000, 0.0000, 0.0000, 0.0000)	(0.4000, 0.6000, 0.4000, 0.5000, 0.3000, 0.4000)	(0.0000, 0.0000, 1.0000, 1.0000, 0.6992, 0.6992)	(0.6000, 0.7000, 0.5000, 0.6000, 0.1000, 0.2000)	(0.0000, 0.0000, 1.0000, 1.0000, 0.7510, 0.7510)
A_4	(0.7453, 0.7453, 0.0000, 0.0000, 0.0000, 0.0000)	(0.4579, 0.6604, 0.3684, 0.4687, 0.2681, 0.3684)	(0.0000, 0.0000, 1.0000, 1.0000, 0.2497, 0.2497)	(0.2000, 0.3000, 0.6000, 0.7000, 0.6000, 0.8000)	(0.0000, 0.0000, 1.0000, 1.0000, 0.7270, 0.7270)
A_5	(0.0000, 0.0000, 0.0000, 0.0000, 0.0000, 0.0000)	(0.2884, 0.5040, 0.4407, 0.5421, 0.3396, 0.4407)	(0.0000, 0.0000, 1.0000, 1.0000, 1.0000, 1.0000)	(0.2732, 0.3743, 0.5241, 0.6257, 0.4160, 0.6350)	(0.0000, 0.0000, 1.0000, 1.0000, 0.7510, 0.7510)

Table S9. The weighted matrix.

Alt.	CC_1	CC_2	CC_5	CC_6	CC_7
A_1	(0.1077, 0.1077, 0.0000, 0.0000, 0.0000, 0.0000)	(0.0842, 0.1432, 0.8908, 0.9158, 0.8568, 0.8908)	(0.0000, 0.0000, 1.0000, 1.0000, 0.0000, 0.0000)	(0.1421, 0.1902, 0.7551, 0.8098, 0.6314, 0.7706)	(0.0000, 0.0000, 1.0000, 1.0000, 0.9941, 0.9941)
A_2	(1, 1, 0.0000, 0.0000, 0.0000, 0.0000)	(0.0842, 0.1432, 0.8908, 0.9158, 0.8568, 0.8908)	(0.0000, 0.0000, 1.0000, 1.0000, 0.6838, 0.6838)	(0.1273, 0.1736, 0.7741, 0.8264, 0.6820, 0.8061)	(0.0000, 0.0000, 1.0000, 1.0000, 0.9943, 0.9943)
A_3	(0.3487, 0.3487, 0.0000, 0.0000, 0.0000, 0.0000)	(0.0479, 0.0842, 0.9158, 0.9356, 0.8908, 0.9158)	(0.0000, 0.0000, 1.0000, 1.0000, 0.9005, 0.9005)	(0.2634, 0.3308, 0.7936, 0.8433, 0.4639, 0.5846)	(0.0000, 0.0000, 1.0000, 1.0000, 0.9949, 0.9949)
A_4	(0.2989, 0.2989, 0.0000, 0.0000, 0.0000, 0.0000)	(0.0571, 0.0985, 0.9085, 0.9298, 0.8813, 0.9085)	(0.0000, 0.0000, 1.0000, 1.0000, 0.6661, 0.6661)	(0.0717, 0.1122, 0.8433, 0.8878, 0.8433, 0.9283)	(0.0000, 0.0000, 1.0000, 1.0000, 0.9943, 0.9943)
A_5	(0.0000, 0.0000, 0.0000, 0.0000, 0.0000, 0.0000)	(0.0322, 0.0651, 0.9243, 0.9429, 0.9015, 0.9243)	(0.0000, 0.0000, 1.0000, 1.0000, 1.0000, 1.0000)	(0.1010, 0.1448, 0.8061, 0.8552, 0.7463, 0.8594)	(0.0000, 0.0000, 1.0000, 1.0000, 0.9949, 0.9949)

Table S10. The border approximation area (BAA) matrix.

	CC_1	CC_2	CC_5	CC_6	CC_7
g_j	(0.0000, 0.0000, 0.0000, 0.0000, 0.0000, 0.0000)	(0.0574, 0.1021, 0.9070, 0.9288, 0.8788, 0.9070)	(0.0000, 0.0000, 1.0000, 1.0000, 1.0000, 1.0000)	(0.1281, 0.1777, 0.7968, 0.8469, 0.6984, 0.8205)	(0.0000, 0.0000, 1.0000, 1.0000, 0.9945, 0.9945)

Supplementary Material B

$A = \{\langle x, T_A(x), I_A(x), F_A(x)\rangle | x \in U, T_A(x), I_A(x), F_A(x) \in [0, 1]\}$ (S1)

where, $T_A(x) = [T_A^L(x), T_A^U(x)]$, $I_A(x) = [I_A^L(x), I_A^U(x)]$, $F_A(x) = [F_A^L(x), F_A^U(x)] \subseteq [0, 1]$, and $0 \leq T_A^U(x)$
$+ I_A^U(x) + F_A^U(x) \leq 3$. Easy to use $x = ([T^L, T^U], [I^L, I^U], [F^L, F^U])$ to represent an interval value neutrosophic number (IVNN) as an element in the IVNS A.

$f_X: E \rightarrow P(U)$ such that $f_X(x) = \varnothing$ if $x \notin X$ (S2)

where the value $fx(x)$ is a set called the x-element of the soft set for all $X \in E$. The sets $fx(x)$ may be arbitrary. Thus, a soft set over U can be represented by the set of ordered pairs.

$F_X = \{(x, f_X(x)): x \in E, f_X(x) \in P(U)\}$ (S3)

$F^c(a) = \{(\text{x}, ([F_{F_a}^L(x), F_{F_a}^U(x)], [1 - I_{F_a}^U(x), 1 - L_{F_a}^L(x)], [T_{F_a}^L(x), T_{F_a}^U(x)])): x \in U, a \in A\}$ (S4)

$$T_{H(e)}(x) = \begin{cases} T_{F(e)}(x), \text{if } e \in A - B \\ T_{G(e)}(x), \text{if } e \in B - A \\ \left[\max\begin{pmatrix}\inf T_A(x), \\ \inf T_B(x)\end{pmatrix}, \max\begin{pmatrix}\sup T_A(x), \\ \sup T_B(x)\end{pmatrix}\right], \text{if } e \in A \cap B \end{cases}$$

$$I_{H(e)}(x) = \begin{cases} I_{F(e)}(x), \text{if } e \in A - B \\ I_{G(e)}(x), \text{if } e \in B - A \\ \begin{bmatrix}\frac{\inf I_A(x) + \inf I_B(x)}{2}, \\ \frac{\sup I_A(x) + \sup I_B(x)}{2}\end{bmatrix}, \text{if } e \in A \cap B \end{cases} \quad \text{(S5)}$$

$$F_{H(e)}(x) = \begin{cases} F_{F(e)}(x), \text{if } e \in A - B \\ F_{G(e)}(x), \text{if } e \in B - A \\ \left[\min\begin{pmatrix}\inf F_A(x), \\ \inf F_B(x)\end{pmatrix}, \min\begin{pmatrix}\sup F_A(x), \\ \sup F_B(x)\end{pmatrix}\right], \text{if } e \in A \cap B \end{cases}$$

$x_{11} \oplus x_{12} = ([T_{11}^L + T_{12}^L - T_{11}^L T_{12}^L, T_{11}^U + T_{12}^U - T_{11}^U T_{12}^U], [I_{11}^L * I_{12}^L, I_{11}^U * I_{12}^U], [F_{11}^L * F_{12}^L, F_{11}^U * F_{12}^U])$ (S6)

$x_{11} \otimes x_{12} = ([T_{11}^L * T_{12}^L, T_{11}^U * T_{12}^U], [I_{11}^L + I_{12}^L - I_{11}^L I_{12}^L, I_{11}^U + I_{12}^U - I_{11}^U I_{12}^U], [F_{11}^L + F_{12}^L - F_{11}^L F_{12}^L, F_{11}^U + F_{12}^U - F_{11}^U F_{12}^U])$ (S7)

$\omega x_{11} = ([1 - (1 - T_{11}^L)^{\omega}, 1 - (1 - T_{11}^U)^{\omega}], [(I_{11}^L)^{\omega}, (I_{11}^U)^{\omega}], [(F_{11}^L)^{\omega}, (F_{11}^U)^{\omega}]$ (S8)

$x_{11}^{\lambda} = ([(T_{11}^L)^{\lambda}, (T_{11}^U)^{\lambda}], [1 - (1 - I_{11}^L)^{\lambda}, 1 - (1 - I_{11}^U)^{\lambda}], [1 - (1 - F_{11}^L)^{\lambda}, 1 - (1 - F_{11}^U)^{\lambda}]$ (S9)

$$S(x_{11}) = [(2+T_{11}^L-I_{11}^L-F_{11}^L)+(2+T_{11}^U-I_{11}^U-F_{11}^U)]/6 \tag{S10}$$

$$H(x_{11}) = [(T_{11}^L+T_{11}^U)-(F_{11}^L+F_{11}^U)]/2 \tag{S11}$$

$$\otimes_{i=1}^{m}(\omega_j x_{ij}) = ([\prod_{i=1}^{m}(T_{ij}^L)^{\omega_j}, \prod_{i=1}^{m}(T_{ij}^U)^{\omega_j}],[1-\prod_{i=1}^{m}(1-I_{ij}^L)^{\omega_j}, 1-\prod_{i=1}^{m}(1-I_{ij}^U)^{\omega_j}], [1-\prod_{i=1}^{m}(1-I_{ij}^L)^{\omega_j}, 1-\prod_{i=1}^{m}(1-I_{ij}^U)^{\omega_j}]) \tag{S12}$$

$$d(x_{11}, x_{12})=\sqrt{(T_{11}^L-T_{12}^L)^2+(T_{11}^U-T_{12}^U)^2+(I_{11}^L-I_{12}^L)^2+(I_{11}^U-I_{12}^U)^2+(F_{11}^L-F_{12}^L)^2+(F_{11}^U-F_{12}^U)^2} \tag{S13}$$

$$D_{\text{IVNSS}}(P, Q) =(\frac{1}{6}\sum_{i=1}^{m}((T_P^L(x_i)-T_Q^L(x_i))^2+(T_P^U(x_i)-T_Q^U(x_i))^2+(I_P^L(x_i))^2-I_Q^L(x_i) + (I_P^U(x_i)-I_Q^U(x_i))^2+ (F_P^L(x_i)-F_Q^L(x_i))^2+(F_P^U(x_i)-F_Q^U(x_i))^2))^{1/2} \tag{S14}$$

$$\xi_{ij} = \begin{cases} \gamma_{ij} - NIT_j & \text{for } \gamma_{ij} > NIT_j \\ 0 & \text{for } \gamma_{ij} \le NIT_j \end{cases} \tag{S15}$$

$$X = \begin{matrix} A_1 \\ A_2 \\ \cdots \\ A_m \end{matrix} \begin{bmatrix} x_{11} & x_{12} & \cdots & x_{1n} \\ x_{21} & x_{22} & \cdots & x_{2n} \\ \cdots & \cdots & \cdots & \cdots \\ x_{m1} & x_{m2} & \cdots & x_{mn} \end{bmatrix} \tag{S16}$$

$$([\tilde{T}_{ij}^L, \tilde{T}_{ij}^U], [\tilde{I}_{ij}^L, \tilde{I}_{ij}^U], [\tilde{F}_{ij}^L, \tilde{F}_{ij}^U]) = \begin{cases} ([T_{ij}^L, T_{ij}^U], [I_{ij}^L, I_{ij}^U], [F_{ij}^L, F_{ij}^U]), h_{ij} \in \text{J}^+ \\ ([F_{ij}^L, F_{ij}^U], [1 - I_{ij}^U, 1 - I_{ij}^L], [T_{ij}^L, T_{ij}^U]), h_{ij} \in \text{J}^- \end{cases} \tag{S17}$$

where J^+ stands for Senefit type indicator, J^- stands for cost type indicator.

$$z_{ij} = ([\acute{T}_{ij}^L, \acute{T}_{ij}^U], [\acute{I}_{ij}^L, \acute{I}_{ij}^U], [\acute{F}_{ij}^L, \acute{F}_{ij}^U])= \omega_j([\tilde{T}_{ij}^L, \tilde{T}_{ij}^U], [\tilde{I}_{ij}^L, \tilde{I}_{ij}^U], [\tilde{F}_{ij}^L, \tilde{F}_{ij}^U]) \tag{S18}$$

$$D = \begin{bmatrix} d_{11} & d_{12} & \cdots & d_{1n} \\ d_{21} & d_{22} & \cdots & d_{2n} \\ \cdots & \cdots & \cdots & \cdots \\ d_{m1} & d_{m2} & \cdots & d_{mn} \end{bmatrix} \tag{S19}$$

$$d_{ij} = \begin{cases} d_H(z_{ij}, g_j), & \text{if } z_{ij} > g_j \\ 0, & \text{if } z_{ij} = g_j \\ -d_H(z_{ij}, g_j), & \text{if } z_{ij} < g_j \end{cases} \tag{S20}$$

where distance measure d_H is defined in Equation (S13).

Disclaimer/Publisher's Note: The statements, opinions and data contained in all publications are solely those of the individual author(s) and contributor(s) and not of MDPI and/or the editor(s). MDPI and/or the editor(s) disclaim responsibility for any injury to people or property resulting from any ideas, methods, instructions or products referred to in the content.
